# Cushing’s syndrome due to ectopic adrenocorticotropin secretion by a parotid carcinoma

**DOI:** 10.20945/2359-3997000000426

**Published:** 2022-01-14

**Authors:** Fernanda F. Antonacio, Guilherme Harada, Rafael S. Vilela, Thais C. Freitas, Jose V. Lima, Luiz Paulo Kowalski, Madson Q. Almeida, Gilberto de Castro

**Affiliations:** 1 Hospital Sírio-Libanês Centro de Oncologia São Paulo SP Brasil Centro de Oncologia, Hospital Sírio-Libanês, São Paulo, SP, Brasil; 2 Hospital Sírio--Libanês Laboratório de Anatomia Patológica São Paulo SP Brasil Laboratório de Anatomia Patológica, Hospital Sírio--Libanês, São Paulo, SP, Brasil; 3 Universidade de São Paulo Faculdade de Medicina Hospital das Clínicas São Paulo SP Brasil Unidade de Suprarrenal, Laboratório de Hormônios e Genética Molecular LIM/42, Serviço de Endocrinologia e Metabologia, Hospital das Clínicas, Faculdade de Medicina da Universidade de São Paulo, São Paulo, SP, Brasil; 4 Irmandade Santa Casa de Misericórdia de São Paulo Laboratório Fleury Serviço de Endocrinologia e Metabologia São Paulo SP Brasil Laboratório Fleury & Serviço de Endocrinologia e Metabologia, Irmandade Santa Casa de Misericórdia de São Paulo, São Paulo, SP, Brasil; 5 Universidade de São Paulo Faculdade de Medicina Hospital das Clínicas São Paulo SP Brasil Departamento de Cirurgia de Cabeça e Pescoço LIM/28, Hospital das Clínicas, Faculdade de Medicina da Universidade de São Paulo, São Paulo, SP, Brasil; 6 Universidade de São Paulo Faculdade de Medicina Instituto do Câncer do Estado de São Paulo São Paulo SP Brasil Serviço de Endocrinologia, Instituto do Câncer do Estado de São Paulo (Icesp), Faculdade de Medicina da Universidade de São Paulo, São Paulo, SP, Brasil

## Abstract

We report a rare case of Cushing’s syndrome in a 37-year-old female who initially presented with localized acinic cell carcinoma of the parotid gland. In January 2014, she underwent a right parotidectomy with facial nerve preservation and adjuvant radiotherapy. In August 2018, she presented a histologically-proven local regional relapse. The patient was considered for salvage surgery with facial nerve sacrifice and remained with no evidence of disease. One year later the patient developed pulmonary dissemination and started to gain weight and developed facial plethora and acne on the face and upper trunk. In a physical examination, the patient presented moon face, buffalo hump, acne and stage 2 hypertension. Biochemical evaluation confirmed ACTH-dependent Cushing’s syndrome. IHC for ACTH in the lung biopsy revealed strong positive staining for ACTH confirming a diagnosis of ectopic ACTH secretion by a metastatic parotid acinic cell carcinoma. Ketoconazole (600 mg/d) was started to treat the CS. In addition, as chemotherapy was initiated to treat the metastatic disease. After the fifth cycle of chemotherapy, ketoconazole was suspended and the patient remained in remission of CS for four months, when CS recurred. A unique feature of this case is related to the clinical CS relapse associated with disease progression, which needed prompt treatment with ketoconazole, resulting in a significant improvement in the patient’s condition. Although rare, should be attentive for possible CS features in patients with high-grade salivary gland carcinomas, since the diagnosis of ectopic secretion of ACTH may significantly impact their management and outcomes.

## INTRODUCTION

Ectopic adrenocorticotropic hormone (ACTH) secretion (EAS) accounts for about 10% to 20% of all Cushing syndrome (CS) cases that are ACTH-dependent (1,2). EAS is associated with several solid tumors, ranging from benign undetectable lesions to generalized metastases. The most common causes of EAS other than small-cell lung carcinoma are neuroendocrine tumors (mainly bronchial), thymic tumors, pancreatic islet tumor, medullary thyroid carcinoma and pheochromocytoma (3).

In rare occasions, malignant salivary gland tumors have been associated with ectopic ACTH production. Unawareness and nonspecific symptoms of paraneoplastic ACTH syndrome have invariably contributed to delayed diagnosis and prevented early therapeutic intervention of this condition (4-6).

In advanced tumors, signs of hypercortisolemia can be masked by a general deterioration of the clinical picture and interpreted as progression of neoplastic disease (7). A definitive diagnosis of EAS requires stringent criteria, including changing of the clinical picture after tumor resection and/or confirming of ACTH immunohistochemical staining (IHC) in the tumor tissue. However, these criteria are not applicable to many of the reported cases of EAS. Tumor resection may not be curative in disseminated tumors and a non-staining biopsy sample does not exclude EAS, since only a subpopulation of cells can in fact secrete ACTH (8).

Only a few cases of ectopic adrenocorticotropin secretion by a parotid carcinoma have been reported in the literature. These cases followed an aggressive clinical course including metastasis and/or high-grade transformation (4-6). Non-recognition of EAS can lead to further morbidity in cancer patients and can also delay cancer treatment and hinder its adherence. 

Therefore, managing EAS efficiently and carefully is the key to achieving adequate cortisol levels and, consequently, better clinical outcomes. That said, this is a case report of a patient with CS secondary to ectopic ACTH secretion due to a parotid adenocarcinoma (IHC positive for ACTH) that was promptly diagnosed and has an excellent outcome both regarding the management of CS and the oncological disease. 

## CASE REPORT

Herein, we report a case of a 37-year-old female patient, diagnosed in early 2014 with acinic cell carcinoma of the parotid gland [immunohistochemistry (IHC) positive for A1/AE3 and DOG1]. Upon initial workup, the tumor was staged as localized disease in the parotid gland with neither lymph node involvement nor distant metastasis (T2N0M0). In January 2014, she underwent a right parotidectomy with facial nerve preservation and adjuvant radiotherapy to the surgical bed in addition to lymph node drainage. In August 2018, she presented a histologically-proven local regional relapse. The patient was considered for salvage surgery with facial nerve sacrifice (2 cm tumor with clear margins), and remained with no evidence of disease until October 2019, when she developed pulmonary dissemination (multiple nodules and mediastinal lymph node involvement). A biopsy of one of the lung lesions was performed, confirming metastatic dissemination, and the pathological features were compatible with relapsed acinic cell carcinoma with high-grade transformation, with no immunoexpression of human epidermal growth factor receptor 2 (HER-2), pan neurotrophin tyrosine receptor kinase (NTRK), or androgen receptor. Next-generation sequencing (FoundationOne CDx, Foundation Medicine, Cambridge, MA, USA) was performed showing cyclin-dependent kinase inhibitor 2A/B (*CDKN2A/B*) and methylthioadenosine phosphorylase (*MTAP*) loss-of-function, D697E mutation in *RB1* and low tumor mutational burden (4mut/Mb). No genomic finding was amenable to targeted therapy.

**Figure 1:**
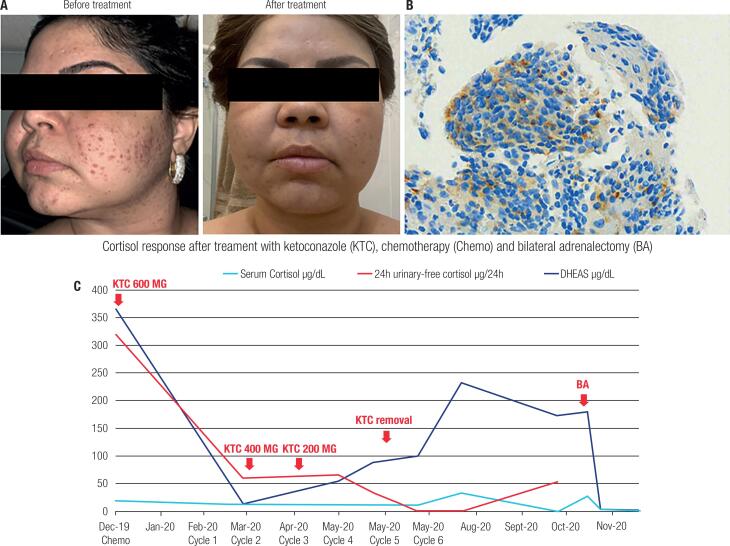
(**A**) Patient presenting plethora, moon face and severe acne at diagnosis. A significant improvement of CS features occurred after treatment with ketoconazole and chemotherapy. (**B**) Immunohistochemistry displaying a strong positive staining for ACTH in a lung metastasis (original magnification ×400, using ACTH Rabbit Polyclonal Antibody. Cell Marquetm, by Ventana BenchMark ULTRA). (**C**) Hormone work up during treatment showing a significant improvement of CS.

In September 2019, the patient started to gain weight and developed facial plethora and acne on the face and upper trunk (Figure 1). In a physical examination, the patient presented moon face, buffalo hump, acne and stage 2 hypertension. Biochemical evaluation confirmed ACTH-dependent Cushing syndrome (CS), with 24h-urinary free cortisol = 598.3 µg/24h (3 to 43 µg), midnight salivary cortisol = 333 ng/dL (<100 ng/dL), cortisol after an overnight 1 mg dexamethasone suppression test = 20.5 µg/dL (<1.8 µg/dL), and adequate dexamethasone blood levels (>130 ng/dL), dehydroepiandrosterone sulfate (DHEAS) = 391 µg/dL (61 to 337 µg/dL) and adrenocorticotropin (ACTH) = 38 pg/mL (7 to 63 pg/mL). Pituitary MRI was normal. A high dose overnight 8 mg dexamethasone suppression test did not show any suppression of cortisol levels, suggesting an ectopic source of ACTH production. We then performed IHC for ACTH in the lung biopsy of a metastatic lesion, which revealed strong positive staining for ACTH (Figure 1), confirming a diagnosis of ectopic ACTH secretion by a metastatic parotid acinic cell carcinoma.  

Ketoconazole (600 mg/d) was started to treat the CS in December 2019. In addition, a combination of carboplatin AUC 5 and paclitaxel (200 mg/m²) was initiated as chemotherapy to treat the metastatic disease in February 2020. After the third cycle of chemotherapy, the ketoconazole dose was reduced to 200 mg/d based on the normalization of 24h-urinary free cortisol, midnight salivary cortisol and DHEAS. After the fifth cycle of chemotherapy, ketoconazole was suspended and the patient remained in remission of CS for four months, when CS recurred. In September 2020, the patient envolve with clinical CS recurrence, confirmed by biochemical evaluation and underwent videolaparoscopic bilateral adrenalectomy remaining in adrenal insufficiency afterwards. 

## DISCUSSION

Ectopic secretion of ACTH by nonpituitary tumors accounts for 5% to 18% of ACTH-dependent CS causes (4). Several tumors have been associated with ectopic ACTH CS. Most cases are caused by neuroendocrine tumors primarily located in the lungs, pancreas, or thymus. Pheochromocytomas and medullary thyroid carcinomas can also produce ACTH and overt clinical CS. ACTH-producing pulmonary small-cell carcinomas are more associated with biochemical hypercortisolism without overt clinical features of CS. Except in some cases of carcinoid tumors, ACTH secretion from malignant ectopic sources is not inhibited by high dexamethasone doses (9). The ectopic source of ACTH is usually confirmed by a petrosal venous sinus catheterization showing absence of a central-to-peripheral ACTH gradient (9).

After the diagnosis of ACTH-dependent hypercortisolism, it is extremely important to locate the focus of ACTH production, considering that Cushing’s disease (pituitary production) is the most common etiology (75%-80%). If the pituitary magnetic resonance imaging is normal or there is a clinical hypothesis for ectopic production of ACTH, dynamic tests can be used in order to determine the etiology (10). The most used tests are the Desmopressin Test (DDAVP), the high dose dexamethasone suppression test and the corticotropin releasing hormone (CRH) test. Corticotrophic pituitary adenomas usually have increased expression of V3 receptors. DDAVP binds to these receptors stimulating ACTH secretion. In Cushing’s disease, stimulation with DDAVP generates an increase in ACTH and cortisol, but 20%-40% of ectopic tumors also have V3 receptors and can respond to this test, limiting its use. The high-dose dexamethasone suppression test (8 mg) is based on the fact that pituitary corticotrophic adenomas respond to the suppressive effects of glucocorticoids and ectopic tumors do not suppress ACTH secretion after this test. A decrease higher than 50% in cortisol levels after overnight 8 mg dexametahose would favor the diagnosis of Cushing’s disease, but 10%-20% of ectopic tumors may also present a decrease of more than 50%, making the response after this test not conclusive for the differential diagnosis (11). Finally, the CRH test is based on the pituitary response to the infusion of CRH, which would be rare in ectopic tumors, but CRH is not easily available and is not routinely performed (12). 

Ectopic ACTH secretion by a parotid carcinoma is a very rare cause of CS. In the case described, the patient developed clinical signs of Cushing’s syndrome in a short period of time (less than 6 months with rapid clinical evolution), which was coincident with the finding of parotid cancer recurrence and lung metastasis. We have performed the overnight high dose 8 mg dexamethasone test which did not show any degree of cortisol suppression, suggesting an ectopic ACTH production. Given that the tissue of the lung metastasis was available and the clinical appearance of Cushing syndrome was very suggestive of ACTH ectopic production, we performed immunohistochemistry for ACTH in the metastatic sample. ACTH staining was strongly positive, confirming the hypothesis of ectopic ACTH production. Therefore, we considered that a petrosal venous sinus catheterization would not be necessary.

Recently, Saluja and cols.(4) reported the case of a 59-year-old man with acinic cell carcinoma of the parotid gland, in whom ACTH ectopic production coincided with disease progression of lung metastasis and mediastinal lymphadenopathy. Unfortunately, the patient presented disease progression and clinical deterioration despite chemotherapy and mifepristone for CS (4). To date, only six cases of CS and ectopic ACTH secretion by a parotid carcinoma have been reported (4-6). ACTH ectopic CS in parotid carcinoma has been associated with high-grade transformation, disease progression and poor prognosis (6).

In contrast to the six cases previously described, in our case chemotherapy produced a partial tumor response, and biochemical normalization with ketoconazole. The patient remained in CS remission without ketoconazole treatment for four months after the 5^th^ cycle of carboplatin and paclitaxel (in a total of six cycles). A unique feature of this case is related to the clinical CS relapse associated with disease progression, which needed prompt treatment with ketoconazole, resulting in a significant improvement in the patient’s condition.

There is a paucity of high-quality data regarding the role of chemotherapy in the metastatic setting of salivary gland carcinomas, either as single agents or in combination regimens, with response rates ranging from 29% to 47% with no clear positive impact on overall survival (13). Although rare, should be attentive for possible CS features in patients with high-grade salivary gland carcinomas, since the diagnosis of ectopic secretion of ACTH may significantly impact their management and outcomes.
